# Molecular and clinical heterogeneity in an Iranian case series of Joubert syndrome

**DOI:** 10.1016/j.ymgmr.2026.101346

**Published:** 2026-08-02

**Authors:** Sheyda Khalilian, Mohadeseh Fathi, Zahra Farbood, Fatemeh Dehghanian, Marjan Masoudi, Soudeh Ghafouri-Fard, Seyed Alireza Dastgheib, Mohammad Miryounesi

**Affiliations:** a Department of Medical Genetics, Shahid Beheshti University of Medical Sciences, Tehran, Iran; b Department of Medical Genetics, Shiraz University of Medical Sciences, Shiraz, Iran

**Keywords:** Joubert syndrome, AHI1, KIAA0586, CSPP1, KIAA0556, CC2D2A, TMEM67

## Abstract

**Background:**

Joubert syndrome (JS) is a rare neurodevelopmental ciliopathy characterized by a distinctive midbrain-hindbrain malformation, manifested by hypotonia, ataxia, developmental delay, and variable multisystem involvement. The condition exhibits marked clinical and genetic heterogeneity, with over 40 genes implicated to date. However, the mutational spectrum and phenotypic presentation of JS in Middle Eastern populations, where consanguinity is prevalent, remain poorly characterized.

**Methods:**

We retrospectively reviewed whole-exome sequencing (WES) data from patients referred to the Comprehensive Medical Genetics Center, Shiraz, Iran, between 2019 and 2024. Patients harboring variants in JS-related genes were identified, and their clinical records were analyzed to establish genotype–phenotype correlations.

**Results:**

A total of 21 cases with variants in JS-associated genes were identified. Neurological manifestations, including developmental delay/intellectual disability, speech defects, seizures, and motor impairment, were the most prevalent findings. Vision problems, renal involvement and polydactyly were also present. Molecular analysis revealed variants in 14 distinct genes, with *AHI1* being the most frequently mutated (3 cases), followed by *KIAA0586*, *CSPP1*, *KIAA0556*, *CC2D2A*, and *TMEM67*. Eight cases carried pathogenic or likely pathogenic variants, while 12 cases had variants of uncertain significance (VUS), highlighting the diagnostic challenges in this genetically heterogeneous condition.

**Conclusion:**

This study expands the mutational landscape of JS in the Iranian population and underscores the utility of WES as a first-tier diagnostic tool for JS and related ciliopathies. The high rate of consanguinity in this cohort likely contributes to the enrichment of autosomal recessive forms. Further functional studies are warranted to clarify the pathogenicity of the numerous VUS identified, which will improve genetic counseling and prenatal decision-making for affected families.

## 1 Introduction

Joubert syndrome (JS; OMIM PS213300) was originally characterized in late 1960s, defined by a set of neurological manifestations including episodic hyperpnea, atypical eye movements, ataxia, intellectual disability, and congenital absence of the cerebellar vermis [1]. In the first few months of life, JS typically presents with hypotonia, irregular breathing patterns, atypical eye movements, and developmental delay. While neurological symptoms are the only manifestation in some patients, others experience multisystem involvement, which may include fibrocystic disease of the kidneys and liver, retinal dystrophy, chorioretinal colobomas, occipital encephalocele, and polydactyly [2]. Because of this broad clinical variability, the condition is now grouped under the umbrella term “Joubert syndrome and related disorders (JSRD)”, which encompasses both Senior-Løken syndrome and COACH syndrome—the latter characterized by colobomas, cognitive impairment (formerly termed “oligophrenia”), ataxia, cerebellar vermis hypoplasia, and hepatic fibrosis [3].

JS is a classic ciliopathy, resulting from defects in the primary cilium, a microtubule-based organelle essential for cellular signaling, sensory perception, and embryonic development [4], [5]. To date, pathogenic variants in over 40 genes have been implicated in JSRD, all encoding proteins that localize to the cilium, basal body, or centrosome [6]. These include *AHI1*, *CC2D2A*, *CSPP1*, *TMEM67*, *RPGRIP1L*, and *KIAA0586*, among others [6]. The encoded proteins are critical for ciliary assembly, maintenance, and function, and their disruption leads to the pleiotropic phenotypes observed in patients. The marked genetic heterogeneity, combined with the variable expressivity and overlapping phenotypes with other ciliopathies poses significant challenges for accurate molecular diagnosis and genotype–phenotype correlation.

Despite advances in next-generation sequencing technologies, the genetic architecture of JS in many populations remains underexplored. Most large-scale studies have focused on European or North American cohorts [7], [8], leaving a gap in our understanding of the mutational spectrum in Middle Eastern populations, where high rates of consanguinity may increase the prevalence of autosomal recessive disorders and reveal novel or rare founder variants. Moreover, the interpretation of variants of uncertain significance (VUS) in JS-related genes remains a major hurdle in clinical practice, often complicating genetic counseling and prenatal decision-making.

In the present study, we screened available whole-exome sequencing (WES) data of patients referred to the Comprehensive Medical Genetics Center, Shiraz, Iran, during 2019–2024 and report the variants within JS-related genes in addition to the clinical findings of 21 cases. We describe the spectrum of pathogenic and likely pathogenic variants identified in this Iranian cohort, highlighting the genetic heterogeneity and the phenotypic diversity observed. Our findings aim to expand the mutational landscape of JS in the Middle Eastern population, facilitate genotype–phenotype correlations, and underscore the utility of WES as a first-tier diagnostic tool for this clinically and genetically heterogeneous group of disorders.

## 2 Methods

### 2.1 Selection of cases

Whole data of recruited cases referred to the Comprehensive Medical Genetics Center, Shiraz, Iran, during 2019–2024 were screened to find those with identified variants in JS-related genes. In this study, we retrospectively reviewed WES data from a consecutive series of patients referred for diverse clinical indications, including unexplained neurodevelopmental disorders, syndromic presentations, and fetal anomalies. Cases with pathogenic or likely pathogenic variants in genes within a curated JS-gene panel were selected for this analysis. Then, the clinical findings of cases were obtained to correlate with genetic findings. Informed consent forms were signed by patients or their legal representatives. All methods were carried out in accordance with relevant guidelines and regulations. Experimental protocols were approved by ethical committee of Shiraz University of Medical Sciences (IR.SUMS.MED.REC.1399.325).

### 2.2 Molecular analyses

Genomic DNA was extracted from peripheral blood using a standard salting-out protocol, and its quality was assessed via NanoDrop 1000 spectrophotometry. Whole-exome sequencing was conducted on an Illumina HiSeq4000 platform with paired-end 101-bp reads, following exonic enrichment using the SureSelectXT2 V6 capture kit. After duplicate removal and quality adjustments, the average depth reached ∼150×, with 85% of targets covered at ≥100×. High-quality reads were aligned to GRCh37/hg19 using BWA [9], and duplicate reads were removed with SAMtools [10]. Variant calling and base recalibration were performed with GATK v4 [11]. Prioritization involved manual filtering against ClinVar, with functional annotation via ANNOVAR [12]. Variants were further evaluated using VarSome, HGMD Professional 2019.4, and dbSNP. Population frequencies (MAF < 0.01) were obtained from gnomAD v2.1.1 and cross-checked with dbSNP, 1000 Genomes, EVS, and gnomAD. In silico pathogenicity was predicted using PolyPhen-2, SIFT, VarSome, and CADD. Segregation analysis was performed in cases where blood samples of other family members were available (Cases 6, 7, 8, 10, 17 and 21).

## 3 Results

### 3.1 Clinical findings

A total of 21 cases with diverse variants in JS-related genes were identified through WES screening. The cohort comprised 10 females (47.6%) and 8 males (38%), with three fetal cases of unspecified sex (14.2%). Ages ranged from 1.5 years to 30 years among live-born patients, with a mean age of 10.6 years. Consanguinity was reported in 19 of 21 families (90.5%), consistent with the autosomal recessive inheritance pattern observed in the majority of cases.

Neurological manifestations were the most prevalent clinical findings, present in all but one patient (Case 19, who presented with isolated renal disease). Developmental delay or intellectual disability was documented in 15 patients (83.3% of live-born patients), while speech defects or delays were noted in 11 cases. Seizures or epilepsy were reported in 9 patients (50% of live-born patients), and motor impairment or motor delay was observed in 6 cases (33.3% of live-born patients). Hypotonia was specifically documented in 3 patients (Cases 3, 5, and 17). Cerebellar atrophy was noted in 2 cases (Cases 2 and 21), and encephalocele was present in 3 fetal cases (Cases 7, 16, and 20). Additional central nervous system findings included hydrocephaly (Case 11), holoprosencephaly (Case 7), and microcephaly (Case 5).

Vision problems were observed in 4 patients, including visual impairment (Cases 2, 4), nystagmus (Case 2), strabismus (Case 15), and ptosis (Case 11). Renal involvement was documented in 3 cases, comprising polycystic kidney disease (Cases 16 and 20) and unspecified renal disease (Case 19). Polydactyly was noted in only one patient (Case 11). No cases of liver disease or retinal dystrophy were reported in this cohort.

Other clinical features included hearing impairment or deafness (Cases 4, 10, 11, and 18), scoliosis (Case 3), muscular atrophy (Case 4), micrognathia and cleft palate (Case 4), failure to thrive (Case 5), coarse facies (Case 5), dysphagia (Case 14), tremor (Case 9), and sparse hairs (Case 13). Family histories were notable for similarly affected siblings or recurrence of neurological symptoms in multiple families (Cases 11, 12, 14, 16, 20, and 21). Table 1 summarizes the clinical data of patients.

**Table 1:** Summary of clinical data of patients (F: Female, M: Male, Y: years).

Case Number	Age	Sex	Consanguinity	Clinical manifestations				
				Vision problem	Kidney disease	Polydactyly	Central Nervous System involvement	Other
1	10 Y	F	Yes	–	–	–	Developmental delay, abnormal gait, moving problem, speech defect	–
2	10 Y	M	Yes	Nystagmus, Visual Impairment	–	–	Global Developmental Delay, Seizure, Ataxia, Cerebellar Atrophy	–
3	8 Y	F	Yes	–	–	–	Developmental delay, Motor delay, Speech delay, Intellectual disability, Hypotonia	Scoliosis
4	10 Y	M	No	Visual Impairment	–	–	Dolichocephaly, Seizure	Muscular Atrophy, Hearing Impairment, Micrognathia, Cleft Palate, Microtestes
5	7 Y	F	Yes	–	–	–	Developmental Delay, Hypotonia, Severe Impairment Of Speech & Movement, Seizure, Microcephaly	Failure to thrive, Coarse facies
6	1.5 Y	F	Yes	–	–	–	Developmental delay, Seizure	–
7	Fetus (18 weeks)	–	Yes	–	–	–	Holoprosencephaly, Encephalocele	–
8	29 Y	M	Yes	–	–	–	Intellectual disability, seizure, speech defect, motor impairment	–
9	17 Y	F	Yes	–	–	–	Seizure, Speech difficulty	Hand and head Tremor
10	1.5 Y	M	Yes	–	–	–	Motor Delay	Hearing Loss, Metabolic Disorder
11	2 Y	F	Yes	Ptosis	–	Polydactyly	Developmental delay, seizure, hydrocephaly, speech defect, motor impairment, mild intellectual disability	There is a history of seizure, strabismus, deafness, paralysis, polydactyly, Intellectual disability, ptosis, and motor impairment in the family.
12	6 Y	F	Yes	–	–	–	Epilepsy	There is history of seizure, anemia, and diabetes in the family.
13	10 Y	M	Yes	–	–	–	Intellectual disability, Motor delay	Sparse hairs
14	2.5 Y	M	No	–	–	–	Developmental delay, Intellectual disability, seizure, speech defect, paralysis, motor impairment	Dysphagia. There is history of cleft lip, speech defect, paralysis, diabetes, and Intellectual disability in the family
15	30 Y	M	Yes	Strabismus	–	–	Mild intellectual disability, unilateral hearing impairment, speech defect, Sixth cranial nerve palsy	–
16	Fetus (14 weeks)	–	Yes	–	Polycystic and enlarged kidney	–	Encephalocele	There is history of Intellectual disability in the family.
17	8 Y	F	Yes	–	–	–	Speech Regression, Hypotonia	–
18	11 Y	F	Yes				Speech defect	Deafness, minor thalassemia
19	7 Y	M	Yes	–	Renal disease	–	–	–
20	Fetus (14 weeks)	–	Yes	–	Polycystic kidney	–	Encephalocele	There is history of encephalocele and polycystic kidney in previous pregnancy.
21	4.5 Y	F	Yes	–	–	–	Severe developmental delay, speech delay, cerebellar atrophy	The younger brother has the same symptoms.

### 3.2 Molecular findings

WES identified variants in 14 distinct genes associated with JSRD. In total, 22 different variants were detected across the 21 cases, all but two being homozygous, consistent with the high rate of consanguinity and autosomal recessive inheritance. As stated in Table 2, variants identified in Cases 12, 13, 17, 19 and 21 were previously reported in the literature. On the other hand, variants identified in Cases1, 10, 11, 16 and 20 had no report in ClinVar.

**Table 2:** Summary of molecular data of patients (AR: autosomal recessive, AD: autosomal dominant, XL: X linked, NR: not reported, Hom: Homozygote, Het: Heterozygote, VUS: variant of uncertain significance).

Case number	Gene	Transcript	Variant	Associated disease	OMIM	Inheritance	Zygosity	ACMG classification	Clinvar	dbSNP rsID	Ref
1	*AHI1*	NM_001134831.2	Exon7 c.349del p.Val117*	Joubert syndrome 3	608629	AR	Hom	Likely Pathogenic	NR	–	–
2	*AHI1*	NM_001134831.2	Exon8 c.917dupA p.Lys307Glufs*3	Joubert syndrome 3	608629	AR	Hom	Pathogenic	Pathogenic	rs1181159671	–
3	*AHI1*	NM_001134831.2	Exon24 c.3421C > T p.Gln1141*	Joubert syndrome 3	608629	AR	Hom	Pathogenic	Pathogenic	–	–
4	*ARL3*	NM_004311.4	Exon3 c.256G > A p.Asp86Asn	Joubert syndrome 35	618161	AR	Hom	VUS	VUS	rs755208642	–
5	*B9D2*	NM_030578.4	Exon4 c.262C > T p.Arg88Cys	Joubert syndrome 34 Meckel syndrome 10	614175	AR	Hom	VUS	VUS	rs781608263	–
6	*CC2D2A*	NM_001378615.1	Exon19 c.2323G > A p.Glu775Lys	COACH syndrome 2 Joubert syndrome 9 Meckel syndrome 6 Retinitis pigmentosa 93	619111 612285 612284 619845	AR	Hom	VUS	VUS	rs751808973	–
7	*CC2D2A*	NM_001378615.1	Exon22 c.2683C > T p.Gln895*	Joubert syndrome 9 Meckel syndrome 6	612285 612284	AR	Hom	Pathogenic	Pathogenic/ Likely Pathogenic	rs764719093	–
8	*CSPP1*	NM_001382391.1	Exon7 c.821 A > G p.His274Arg	Joubert syndrome 21	615636	AR	Hom	VUS	VUS	rs377496424	–
9	*CSPP1*	NM_001382391.1	Exon24 c.3275C > T p.His274Arg	Joubert syndrome 21	615636	AR	Hom	VUS	VUS	rs768603321	–
10	*KIAA0556*	NM_015202.5	Exon9 c.1044G > T p.Gln348His	Joubert syndrome 26	616784	AR	Hom	VUS	NR	–	–
11	*KIAA0556*	NM_015202.5	Exon23 c.4259G > T p.Gly1420Val	Joubert syndrome 26	616784	AR	Hom	VUS	NR	rs1265663435	–
12	*KIAA0586*	NM_001329943.3	Exon1 c.38del p.Lys13Argfs*6	Joubert syndrome 23 Short-rib thoracic dysplasia 14 with polydactyly	616490 616546	AR	Hom	Pathogenic	Pathogenic/ Likely Pathogenic	rs745949846	[13]
13	*KIAA0586*	NM_001329943.3	Exon5 c.428delG p.Arg131Lysfs*4	Joubert syndrome 23 Short-rib thoracic dysplasia 14 with polydactyly	616490 616546	AR	Hom	Pathogenic	Pathogenic/ Likely Pathogenic	rs534542684	[14]
14	*OFD1*	NM_003611.3	Exon12 c.1196C > T p.Ser399Phe	?Retinitis pigmentosa 23 Joubert syndrome 10 Orofaciodigital syndrome I Simpson-Golabi-Behmel syndrome, type 2	300424 300804 311200 300209	XL	Hemi	VUS	VUS	rs748851759	–
	*CACNA1A*	NM_001127222.2	Exon46 c.6565G > A p.Asp2189Asn	Developmental and epileptic encephalopathy 42 Episodic ataxia, type 2 Migraine, familial hemiplegic, 1 Migraine, familial hemiplegic, 1, with progressive cerebellar ataxia Spinocerebellar ataxia 6	617106 108500 141500 141500 183086	AD AD AD AD AD	Het	VUS	VUS	–	–
15	*RPGRIP1L*	NM_015272.5	Exon17 c.2474 A > G p.His825Arg	COACH syndrome 3 Joubert syndrome 7 Meckel syndrome 5	619113 611560 611561	AR	Hom	VUS	VUS	rs752772082	–
16	*TCTN1*	NM_001082538.3	c.*1 + 1G > A	Joubert syndrome 13	614173	AR	Hom	Likely Pathogenic	NR	rs1012449234	–
17	*TCTN2*	NM_024809.5	c.1232-1G > A	Meckel syndrome 8 Joubert syndrome 24	613885 616654	AR	Hom	Likely Pathogenic	Pathogenic	rs863225425	[15]
18	*TCTN3*	NM_015631.6	Exon1 c.181C > A p.Pro61Thr	Joubert syndrome 18 Orofaciodigital syndrome IV	614815 258860	AR	Hom	VUS	VUS	rs999127884	–
	*PJVK*	NM_001042702.5	Exon7 c.1022 T > G p.Val341Gly	Deafness, autosomal recessive 59	610220	AR	Hom	VUS	VUS	–	–
19	*TMEM67*	NM_153704.6	Exon17 c.1700 A > G p.Tyr567Cys	COACH syndrome 1 Joubert syndrome 6 Meckel syndrome 3 Nephronophthisis 11	216360 610688 607361 613550	AR	Hom	VUS	VUS	rs148726767	[16]
20	*TMEM231*	NM_001077418.3	Exon3 c.371 T > A p.Leu124His	Joubert syndrome 20 Meckel syndrome 11	614970 615397	AR AR	Hom	VUS	NR	–	–
21	*TMEM67*	NM_153704.6	Exon7 c.653G > C p.Gly218Ala	COACH syndrome Joubert syndrome 6 Meckel syndrome 3	216360 610688 607361	AR	Het	VUS	VUS/Benign	rs202036490	[17]
			c.958 A > T p.Ser320Cys				Het	Likely Benign	Conflict	rs111619594	[18]

Case 14 carried a hemizygous variant in *OFD1* (X-linked) and a heterozygous variant in *CACNA1A* (autosomal dominant), while Case 21 carried two distinct heterozygous variants in *TMEM67*.

The most frequently mutated gene was *AHI1*, with variants identified in three cases (Cases 1–3), all of which were homozygous loss-of-function mutations: c.349del (p.Val117*), c.917dupA (p.Lys307Glufs*3), and c.3421C > T (p.Gln1141*). These were classified as pathogenic or likely pathogenic by ACMG criteria and were associated with JS type 3.

Variants in *KIAA0586* were identified in two cases (Cases 12 and 13), both homozygous frameshift mutations (c.38del, p.Lys13Argfs*6 and c.428delG, p.Arg131Lysfs*4) classified as pathogenic and associated with JS type 23. Two cases (Cases 8 and 9) carried homozygous missense variants in *CSPP1* (c.821 A > G, p.His274Arg and c.3275C > T, p.His274Arg), both classified as VUS and associated with JS type 21. Similarly, two cases (Cases 10 and 11) harbored homozygous missense variants in *KIAA0556* (c.1044G > T, p.Gln348His and c.4259G > T, p.Gly1420Val), both classified as VUS and associated with JS type 26.

Two cases (Cases 6 and 7) carried variants in *CC2D2A*, with Case 7 harboring a pathogenic nonsense mutation (c.2683C > T, p.Gln895*) and Case 6 carrying a VUS missense variant (c.2323G > A, p.Glu775Lys). Both were associated with JS type 9 and COACH syndrome.

Variants in *TMEM67* were identified in two cases (Cases 19 and 21). Case 19 carried a homozygous VUS missense variant (c.1700 A > G, p.Tyr567Cys), while Case 21 carried two heterozygous variants: c.653G > C (p.Gly218Ala) classified as VUS and c.958 A > T (p.Ser320Cys) classified as likely benign. These were associated with JS type 6 and COACH syndrome.

Splice-site variants were identified in two cases: Case 16 (TCTN1, c.*1 + 1G > A, likely pathogenic) and Case 17 (TCTN2, c.1232-1G > A, likely pathogenic). These were associated with JS types 13 and 24, respectively.

Additional variants were identified in *ARL3* (Case 4, c.256G > A, p.Asp86Asn, VUS), B9D2 (Case 5, c.262C > T, p.Arg88Cys, VUS), *RPGRIP1L* (Case 15, c.2474 A > G, p.His825Arg, VUS), and *TMEM231* (Case 20, c.371 T > A, p.Leu124His, VUS). Case 14 carried a hemizygous VUS in *OFD1* (c.1196C > T, p.Ser399Phe) and a heterozygous VUS in *CACNA1A* (c.6565G > A, p.Asp2189Asn), the latter associated with autosomal dominant neurological conditions. All VUS were confirmed to have a minor allele frequency below 0.01 in population databases.

Overall, 8 of the 21 cases (38%) carried variants classified as pathogenic or likely pathogenic, while 12 cases (57.14%) carried VUS, and a single case was compound heterozygote for a VUS and a likely benign variant. This data underscores the diagnostic challenges inherent in genetically heterogeneous conditions such as JS. Table 2 summarizes he molecular data of cases.

## 4 Discussion

JS is primarily inherited in an autosomal recessive manner, although a small number of X-linked cases have also been documented. To date, pathogenic variants in more than 40 genes have been implicated in JS, contributing to the wide spectrum of phenotypic variability observed among affected individuals [6]. In the current study, we provided a list of JS-related variants in a population of Iranian patients with diverse clinical findings mostly compatible with JSRD. Previous studies of JSRD are limited, including those describing clinical and imaging findings of single cases [19], [20], [21], or case series [22], mostly without genetic confirmation. Among the very limited genetic data of JS among Iranian patients are c.1064 T > G (p.L355R) variant of the *AHI1* gene [23], and c.725 A > G (p.Asn242Ser) in the *TMEM67* gene [24]. Notably, the latter has been identified in multiple Iranian families with JS [24].

In this study, we present data of variants within JS-related genes. Data corresponds to 21 Iranian patients, including both fetus and live-born ones. Our retrospective approach, using WES data from a clinically diverse cohort, means that while some cases were initially suspected to have JS based on classic features, others may represent incidental or targeted diagnoses identified through WES for broader phenotypes, such as isolated renal disease or fetal anomalies. This highlights the importance of WES as a diagnostic tool for clinically heterogeneous conditions. In addition, our findings underscore the remarkable clinical and genetic heterogeneity of this condition and contribute to the expanding knowledge of the mutational spectrum in Middle Eastern populations, where consanguineous unions are common.

The clinical presentations observed in our cohort were consistent with the well-established features of JS, with neurological manifestations being the most prominent. As expected, developmental delay or intellectual disability was documented in the majority of patients; and speech defects and seizures were detected in a considerable percentage of cases. These findings align with previous large-scale studies, such as the cohort of 100 patients reported by Vilboux et al. (2017), where developmental delay and neurologic findings were dominant features [25]. The presence of cerebellar atrophy in two of our cases (Cases 2 and 21) further supports the characteristic hindbrain involvement that defines the molar tooth sign on neuroimaging, although imaging data were not systematically available for all patients in this retrospective series.

Multisystem involvement, while less frequent in our cohort compared to some Western studies, was still observed. Vision problems, including visual impairment, nystagmus, strabismus, and ptosis were lower than the reported prevalence of retinal dystrophy reported in some series [25]. Renal disease, including polycystic kidneys, was documented in patients, consistent with the known association between JS and nephronophthisis-like renal ciliopathies. Notably, polydactyly was observed in only one patient (Case 11), a lower frequency than the 8–16% reported in previous studies [1], [26], [27]. The absence of liver disease and retinal dystrophy in our cohort may reflect the specific genetic etiologies prevalent in this population, as well as the relatively young age of some patients, as certain manifestations (e.g., retinal degeneration and hepatic fibrosis) may present later in life.

The high rate of consanguinity in our cohort is striking and consistent with the autosomal recessive inheritance pattern of the majority of JS genes. This finding mirrors reports from other Middle Eastern and South Asian populations, where founder effects and endogamy have been shown to increase the prevalence of rare recessive disorders. The predominance of homozygous variants in our molecular data further corroborates this observation.

From a molecular perspective, we identified variants in 14 different genes, reflecting the extensive genetic heterogeneity of JSRD. *AHI1* was the most frequently mutated gene, accounting for three cases (14.28%), all with homozygous loss-of-function variants (nonsense or frameshift). This is consistent with the known role of *AHI1* as one of the major JS genes, particularly among Iranian patients [23], [28]. Indeed, all three *AHI1*-positive cases in our series presented with predominantly neurological features and no renal or liver disease, suggesting a probable genotype–phenotype correlation for this gene.

Pathogenic variants in *KIAA0586* were identified in two cases, both with frameshift mutations leading to protein truncation. This gene has been implicated in JS type 23 and short-rib thoracic dysplasia, and our findings add to the growing body of evidence that *KIAA0586* mutations are a recurrent cause of JSRD [29]. Similarly, variants in *CC2D2A*, including a pathogenic nonsense mutation (Case 7) and a VUS missense variant (Case 6), were observed in two patients. *CC2D2A* is known to cause a spectrum of ciliopathies, including JS type 9, COACH syndrome, and Meckel syndrome, consistent with the severe fetal phenotypes (encephalocele, holoprosencephaly) observed in our Case 7 [30], [31].

Notably, a substantial proportion of our cases carried VUS according to ACMG criteria. This highlights a major challenge in the molecular diagnosis of JS, where many identified variants are novel or rare missense changes with limited functional evidence. The VUS included missense variants in genes such as *ARL3*, *B9D2*, *CSPP1*, *KIAA0556*, *RPGRIP1L*, *TCTN3*, *TMEM67*, and *TMEM231*. Several of these genes are well-established JS loci, but the pathogenicity of the specific variants identified requires further investigation through functional studies, segregation analysis within families, and cross-referencing with large-scale population databases. The presence of VUS complicates genetic counseling, particularly in prenatal settings, and underscores the need for integrated approaches combining in silico prediction, family segregation, and, where possible, cellular or animal models to establish definitive genotype–phenotype correlations.

Case 14 is noteworthy for carrying a hemizygous variant in *OFD1* (X-linked) and a heterozygous variant in *CACNA1A* (autosomal dominant). *OFD1* mutations are associated with JS type 10, orofaciodigital syndrome I, and retinitis pigmentosa [32], while *CACNA1A* variants cause a range of neurological conditions, including episodic ataxia and developmental encephalopathies [33]. The dual variants in this patient may contribute to the complex phenotype observed, including muscular atrophy, hearing impairment, cleft palate, and micrognathia, though the clinical significance of each variant remains uncertain due to their VUS status.

Case 21, with two heterozygous variants in *TMEM67* (one VUS and one likely benign), illustrates the diagnostic complexity in autosomal recessive disorders. The presence of a deep intronic mutation, a large deletion/duplication not captured by WES, or a second variant in another gene (digenic inheritance) should be considered in this patient.

The predominance of VUS in this cohort raises important questions about the interpretation of WES data in highly consanguineous populations. While homozygosity mapping can prioritize candidate variants, the abundance of rare population-specific variants can lead to false-positive associations. Therefore, rigorous filtering, family segregation, and functional validation are essential to distinguish pathogenic mutations from benign rare polymorphisms. This complexity can be reduced by using of additional technologies, such as RNA sequencing (to assess splicing effects) or targeted functional assays in patient-derived cells.

Several points should be considered in interpretation of our results. First, in the current study, we tended to find possible variants associated with JS among Iranian population. This data can be used for genetic counseling purposes. Second, the genetic architecture of JS in the Iranian population may involve novel genes or non-coding regulatory variants not captured by standard exome sequencing. Third, the clinical diagnosis of JS may have been more challenging in some cases without definitive neuroimaging evidence of the molar tooth sign, which was not uniformly available. Nevertheless, the identification of a molecular correlate in one-third of cases represents a valuable contribution to the clinical management and genetic counseling of affected families, and underscores the utility of WES as a first-tier diagnostic tool for genetically heterogeneous disorders.

An important conceptual framework that has emerged from recent genetic and clinical studies is the view of Joubert syndrome as part of a continuous spectrum of ciliopathies, rather than a distinct entity [34]. This spectrum spans from the most severe, often embryonic-lethal Meckel syndrome, through JS with variable multisystem involvement, to the milder, predominantly renal phenotype of nephronophthisis [34]. The near-complete genetic overlap among these conditions—whereby pathogenic variants in the same genes (e.g., *CC2D2A*, *TMEM67*, *RPGRIP1L*, *TCTN2*, and *CSPP1*) can give rise to phenotypes along this continuum—supports this paradigm and explains the remarkable pleiotropy observed clinically.

Our cohort exemplifies this spectrum concept. At the severe end, fetal Cases 7 (*CC2D2A* nonsense variant), 16 (*TCTN1* splice-site variant), and 20 (*TMEM231* VUS) presented with encephalocele, and in Case 7, holoprosencephaly—features classically associated with Meckel syndrome, a condition often prenatally lethal. Conversely, Case 19, who carried a homozygous VUS in *TMEM67*, presented with isolated renal disease without any neurological manifestations, aligning more closely with a nephronophthisis-like phenotype. This is noteworthy, as *TMEM67* mutations are among the most frequently implicated in the renal end of the ciliopathy spectrum. The remaining cases, with their predominant neurological features (developmental delay, ataxia, seizures) and variable presence of renal, ocular, or skeletal involvement, occupy the intermediate JS portion of the spectrum. This distribution of phenotypes within a single cohort, linked by variants in genes known to contribute to the entire ciliopathy continuum, provides strong clinical support for the spectrum model. Recognizing JS as a spectrum condition has important implications for clinical management, as it necessitates comprehensive, multi-system surveillance for patients and their families, and underscores the need for careful genetic counseling that accounts for the potential for variable expressivity and the risk of more severe phenotypes in subsequent pregnancies.

The consanguinity rate in our cohort (90.5%) exceeds the Iranian national average (∼38–40%), reflecting selection bias inherent to a tertiary referral center that attracts consanguineous families seeking diagnoses for recessive disorders. While this enrichment facilitates the detection of homozygous variants, it limits generalizability to non-consanguineous populations. Nonetheless, our findings are relevant to other Middle Eastern and South Asian populations where consanguinity is common.

Several limitations of this study should be acknowledged. First, its retrospective nature and the lack of systematically collected neuroimaging data (e.g., MRI to confirm the molar tooth sign) limited our ability to establish the classic radiological diagnosis in all cases. Second, the absence of parental segregation studies for many variants precluded definitive confirmation of inheritance and pathogenicity. Third, functional validation of VUS was not performed, which would be necessary to definitively classify these variants. Finally, the relatively small sample size limits the generalizability of our findings to the broader Iranian or Middle Eastern population.

Despite these limitations, this study provides valuable insights into the possible genetic causes of JS in Iran. The high prevalence of consanguinity and the identification of variants in genes such as *AHI1* and *KIAA0586* highlight the importance of targeted genetic screening in this population. Furthermore, our findings emphasize the need for comprehensive, multi-disciplinary approaches to the diagnosis and management of JS, involving clinical geneticists, neurologists, nephrologists, ophthalmologists, and genetic counselors.

In conclusion, our case series expands the mutational spectrum of JS in the Middle Eastern population and confirms the utility of WES as a powerful diagnostic tool for this heterogeneous group of disorders. The identification of numerous VUS underscores the ongoing challenges in variant interpretation and the critical need for functional studies to resolve these uncertainties. Future research should focus on large-scale genomic studies, including whole-genome sequencing and transcriptomic analyses, to identify novel disease genes and regulatory variants, and to establish robust genotype–phenotype correlations that will ultimately improve genetic counseling, prenatal diagnosis, and personalized management for patients and families affected by JS.

## CRediT authorship contribution statement

**Sheyda Khalilian:** Formal analysis, Data curation. **Mohadeseh Fathi:** Formal analysis, Data curation. **Zahra Farbood:** Investigation. **Fatemeh Dehghanian:** Investigation. **Marjan Masoudi:** Investigation. **Soudeh Ghafouri-Fard:** Writing – review & editing, Writing – original draft, Supervision. **Seyed Alireza Dastgheib:** Methodology. **Mohammad Miryounesi:** Supervision.

## Declaration of competing interest

Authors declare no conflicts of interests.

## Data Availability

No data was used for the research described in the article.
